# Supplementary material for: Optical information transmission through complex scattering media with optical-channel-based intensity streaming
Source: Nat Commun. 2021 Apr 23;12:2411. doi: 10.1038/s41467-021-22692-1 (PMC8065103; doi:10.1038/s41467-021-22692-1)
Supplement: Supplementary file 1 — Supplementary Information [file 41467_2021_22692_MOESM1_ESM.pdf]

Optical information transmission through complex scattering media with optical-channel-based intensity streaming

Supplementary Information

Haowen Ruan[1,†,*], Jian Xu[1,†,*], and Changhuei Yang[1,*]

[1]Department of Electrical Engineering, California Institute of Technology, Pasadena, CA, 91125

[†]These authors contributed equally to this work.

[*]Contact emails: hruan@caltech.edu, jxxu@caltech.edu, or chyang@caltech.edu

Supplementary Figures

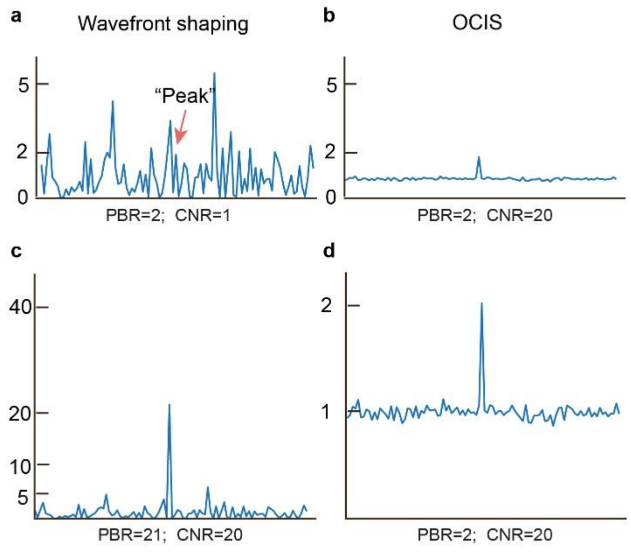

Supplementary Figure 1. Simulation results for comparison of the metrics, peak to background ratio (PBR) and contrast of noise ratio (CNR), which evaluate the quality of a focus pattern. **(a)** In optical wavefront shaping, at low PBR, e.g. PBR=2, the peak is immersed into the background of fully developed speckles, where the standard deviation of the speckle intensity is the same as its mean. In this case, CNR=PBR-1=1. **(b)** For the same PBR, the time-averaged pattern created by OCIS shows a prominent peak as the variation of the background is much lower, resulting in a higher CNR, e.g. CNR=20 (~1000 controllable modes). **(c)** To obtain the same CNR as the pattern formed by OCIS, the PBR of the focus formed by the wavefront shaping techniques needs to increase to 21. **(d)** The pattern in (b) is rescaled to help visually compare to the pattern in (c). As shown in (c) and (d), as long as the CNR is the same, the visibility of the peak is very similar although they have a very different PBR. Therefore, CNR is a more useful metric for OCIS.

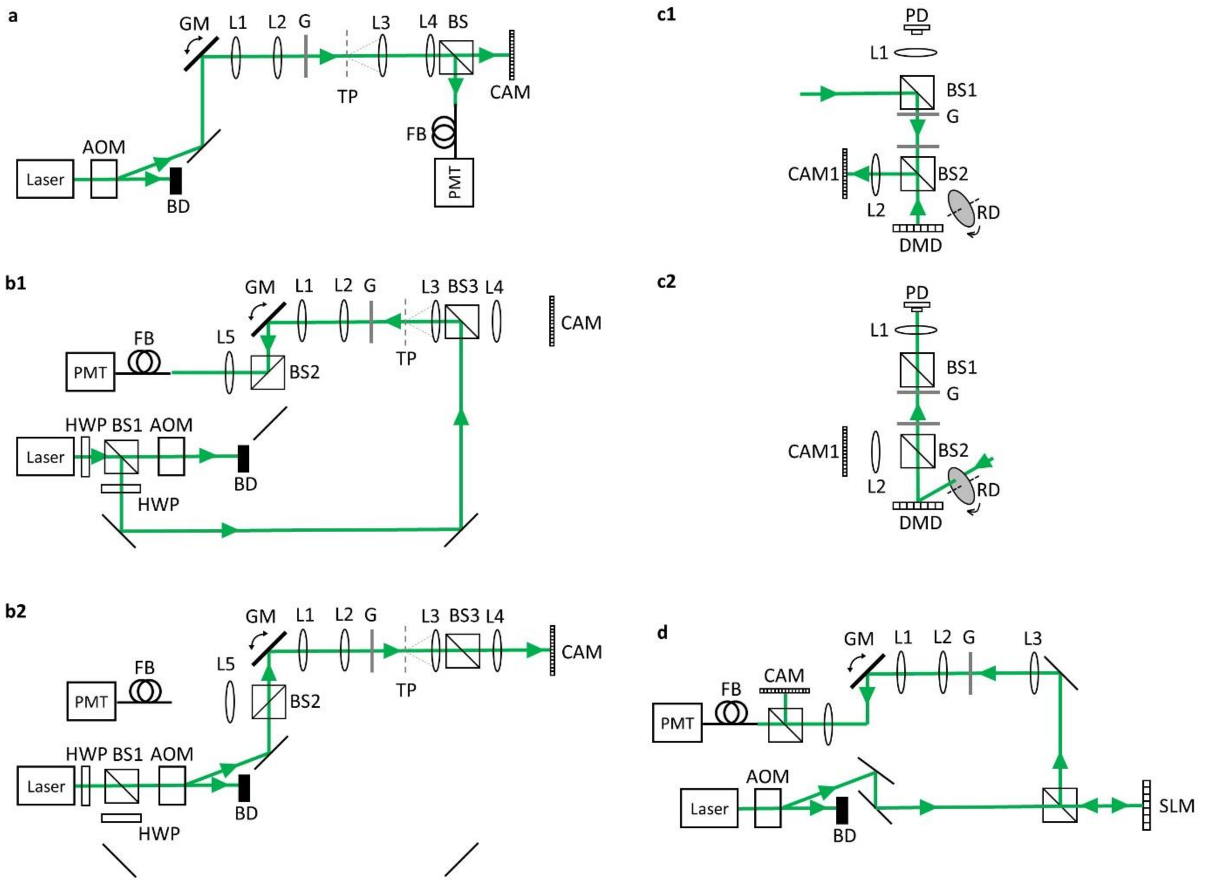

Supplementary Figure 2. Experimental Setups. (a) Feedback based OCIS setup. **(b)** Optical intensity transpose setup. **(b1)** recording; **(b2)** playback. **(c)** Setup for optical information transmission through scattering media. **(c1)** Identifying communication channels; **(c2)** Transmitting information through communication channels. **(d)** Setup for direct imaging through scattering media. Abbreviations: AOM, acousto-optic modulator; BD, beam dump; BS, beam splitter; CAM, camera; DMD, digital micro-mirror device; FB, fiber; G, ground glass diffuser; GM, galvanometer mirror; HWP, half wave plate; L, lens; PD, photodetector; PMT, photomultiplier tube; RD, rotating diffuser; TP, target plane.

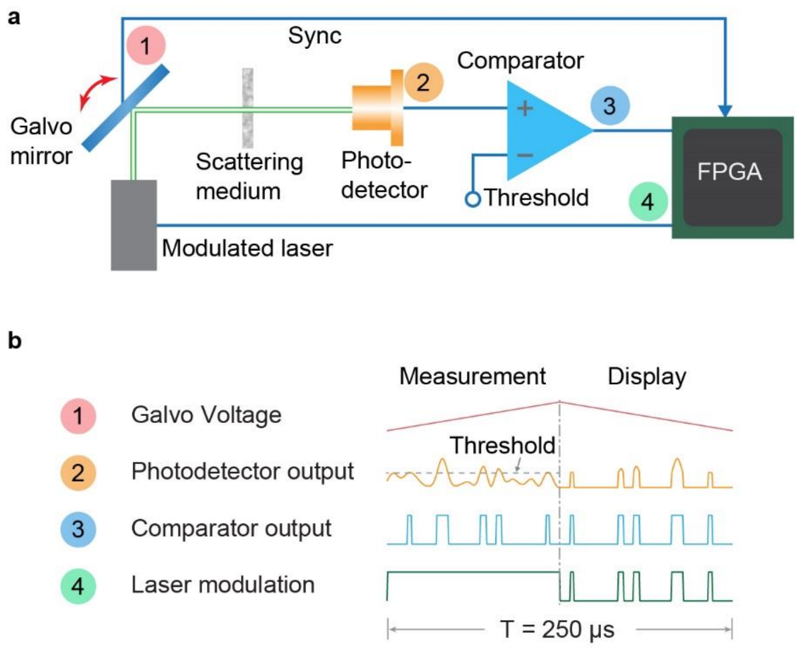

Supplementary Figure 3. (a) Simplified system setup. As the galvo mirror scanned forward, the photodetector measured the temporal signal which was then binarized by the comparator. An FPGA, which was synchronized with the galvo mirror, received the digital signal and output the time-reversed signal that modulated the laser as the galvo mirror scanned backward. **(b)** Illustration of the signals during one galvo mirror round trip.

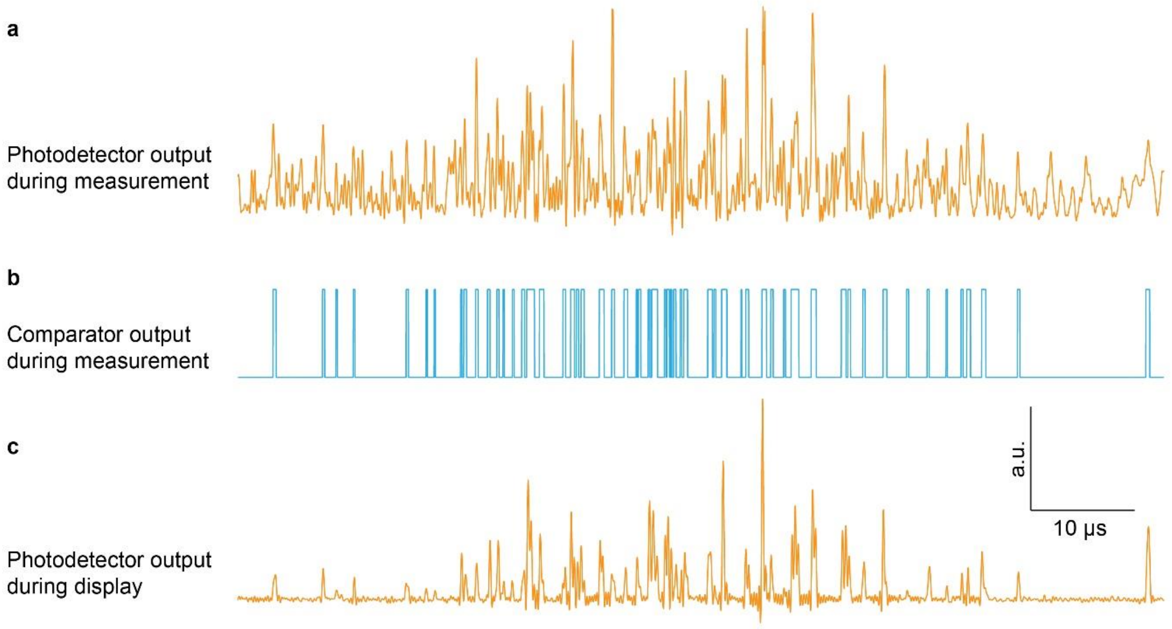

Supplementary Figure 4. Experimental signal traces from the feedback-based OCIS. (a) A raw signal output from the photodetector during measurement. **(b)** A binarized signal output from the comparator during measurement. **(c)** A photodetector output signal during display. To provide a clearer visual comparison, we time-reversed this output signal again to match the timing.

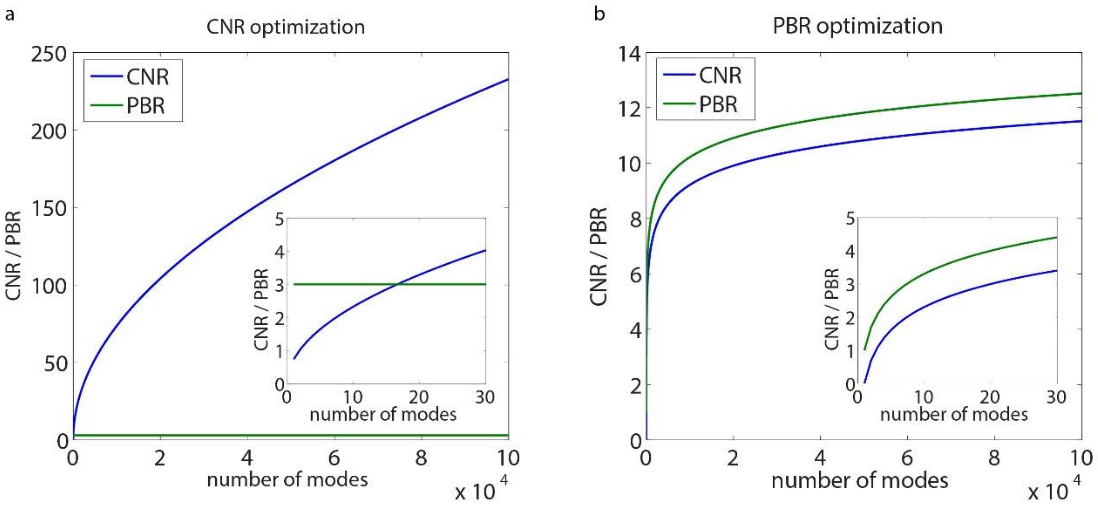

Supplementary Figure 5. CNR and PBR as a function of number of controllable modes. (a) CNR optimization mode based on Supplementary Equation (7) and Supplementary Equation (8). **(b)** PBR optimization mode based on Supplementary Equation (9) and Supplementary Equation (10).

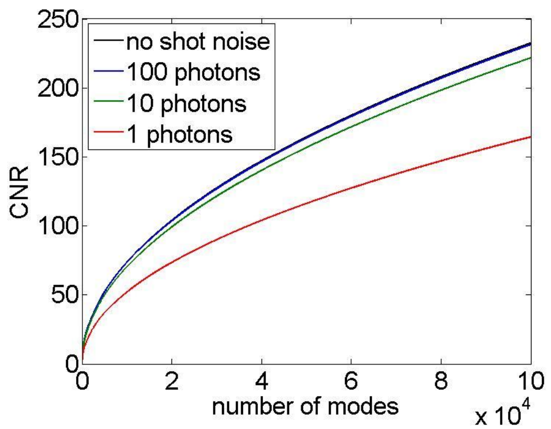

Supplementary Figure 6. CNR as a function of number of modes including shot noise. CNR enhancement plots with different numbers of photons per speckle at the background.

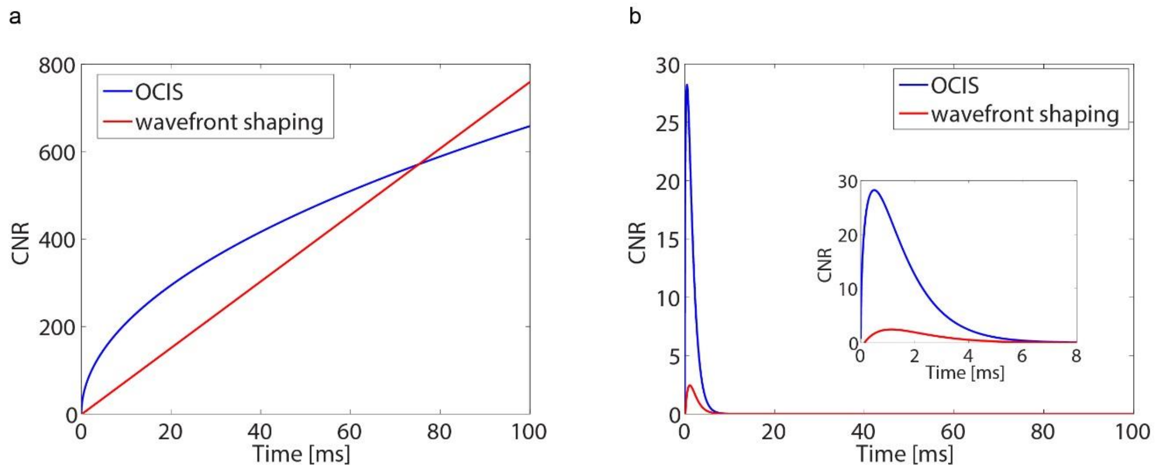

Supplementary Figure 7. Comparison of feedback based OCIS and feedback based wavefront shaping on the CNR as a function of operation time. (a) with static samples. **(b)** with samples of 1 ms decorrelation time in a negative exponential decorrelation model. See Supplementary Note 2 for detailed analysis.

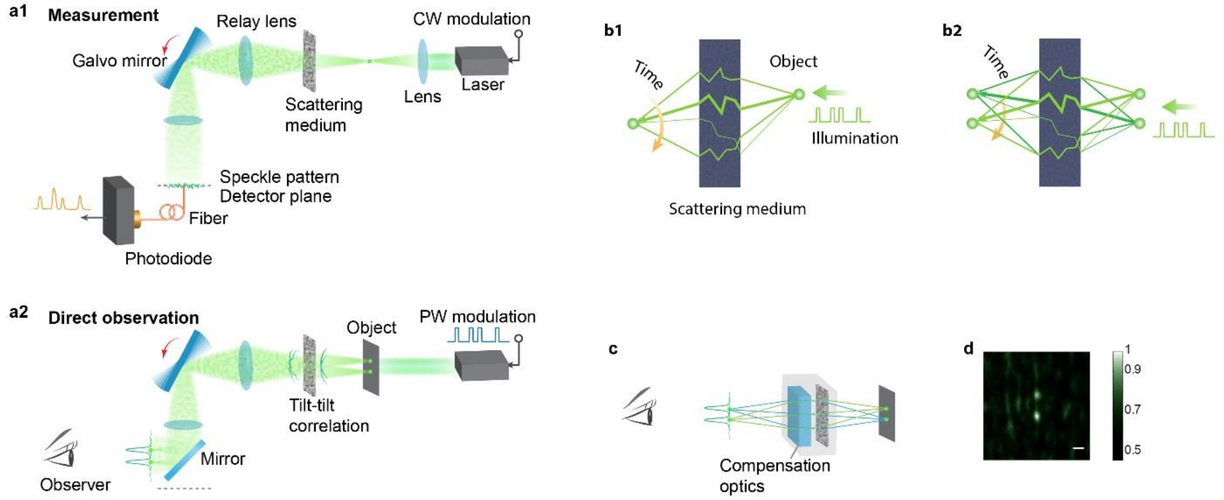

Supplementary Figure 8. Direct imaging through a thin scattering medium with OCIS. (a) Experimental setup. This procedure can be separated into two steps. **(a1)** Measurement. This procedure is the same as the recording of the intensity reflection method described above. An optical spot was created on the target plane and a binarized speckle intensity is measured sequentially on the detector plane during a sweep of the galvo mirror. **(a2)** Direct observation. By using this signal to modulate the laser that illuminates a transmission object on the target plane, one can directly observe the object as the galvo mirror synchronizes with the modulated illumination. The method utilizes the angular memory effect of the thin scattering medium, where a tilted optical field incident to the thin scattering medium results in a tilted optical field on the other side. Therefore, the measured signal is also applicable to the neighboring points

and enables direct observation of the object with only one measurement. **(b)** Optical diagram of the imaging process. **(b1)** Light from an object couples to different high-throughput channels over time and the transmitted light is directed to a spot to form a PSF of the imaging system. **(b2)** Based on the optical memory effect, a neighboring spot within the memory effect range also forms an image at the imaging plane. **(c)** Equivalently, OCIS and the scattering medium serves as an imaging system and one can see through the scattering medium directly. **(d)** An image of the object was formed through the scattering medium and captured by a camera on the detector plane. Scale bar: 10 μm.

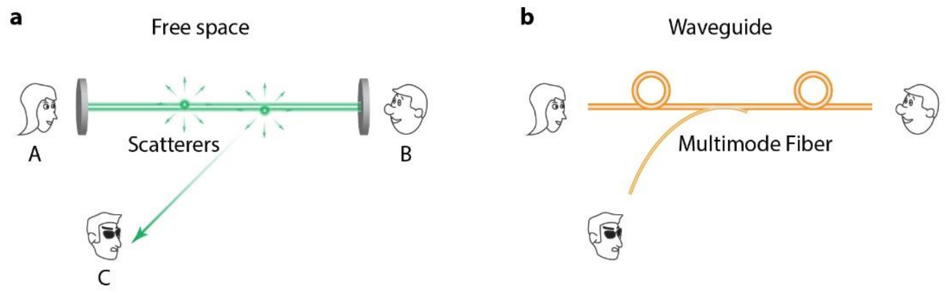

Supplementary Figure 9. Optical information coupled out of the communication chain. (a) In free space, scatterers spread light to other directions. **(b)** In waveguide geometry, light can be coupled out of an optical fiber.

Supplementary Notes

Supplementary Note 1 | Mathematical derivation of CNR and PBR

OCIS optical spot

Here we quantitatively evaluate the performance of OCIS techniques. Assuming the instantaneous speckle patterns are fully developed[1], the speckle intensity follows an exponential distribution with mean μ and standard deviation $\sigma = \mu$. The shot noise effect will be considered in the next section. The probability density function is given by

$$P(I) = \frac{1}{\mu}\exp\left(-\frac{I}{\mu}\right), \tag{1}$$

where μ is the mean intensity of the speckle pattern. Then, α, the portion of patterns in which the intensity value of the pixel of interest is higher than a threshold I_t is given by

$$\alpha = \int_{I_\mathrm{t}}^{\infty} \frac{1}{\mu}\exp\left(-\frac{I}{\mu}\right) dI = \exp\left(-\frac{I_\mathrm{t}}{\mu}\right). \tag{2}$$

The mean intensity of the pixel of interest among these patterns is therefore given by

$$\overline{I_\mathrm{p}} = \int_{I_\mathrm{t}}^{\infty} I \times \frac{P(I)}{\alpha} dI . \tag{3}$$

Substituting Supplementary Equations (1) and (2) into Supplementary Equation (3) leads to

$$\overline{I_\mathrm{p}} = \mu + I_\mathrm{t} . \tag{4}$$

If our system captures N independent speckle patterns in total, the number of selected patterns is then approximately αN. Since the OCIS sums up all the selected patterns, the peak intensity of the resultant pattern on average is given by $\overline{I_\mathrm{sp}} = \alpha N \overline{I_\mathrm{p}}$, while the mean and standard deviation of the background of the resultant pattern is given by $\overline{I_\mathrm{sb}} = \alpha N \mu$ and $\sigma_\mathrm{sb} = \sqrt{\alpha N}\sigma = \sqrt{\alpha N}\mu$. The contrast-to-noise ratio (CNR) is given by

$$\mathrm{CNR_P} = \frac{\overline{I_\mathrm{sp}} - \overline{I_\mathrm{sb}}}{\sigma_\mathrm{sb}} = \sqrt{\alpha N}\frac{I_\mathrm{t}}{\mu} = \sqrt{N}\exp\left(-\frac{I_\mathrm{t}}{2\mu}\right)\frac{I_\mathrm{t}}{\mu} . \tag{5}$$

The PBR of OCIS is given by

$$\mathrm{PBR_P} = \frac{\overline{I_{\mathrm{sp}}}}{\overline{I_{\mathrm{sb}}}} = \frac{\alpha N \overline{I_{\mathrm{p}}}}{\alpha N \mu} = \frac{\mu + I_{\mathrm{t}}}{\mu} = 1 + \frac{I_{\mathrm{t}}}{\mu}. \tag{6}$$

From Supplementary Equation (5) and (6), we find that both CNR and PBR are functions of intensity threshold I_{t} that we choose. Therefore, by selecting a proper threshold, we can optimize the CNR or PBR accordingly. Here we analyze the solutions for CNR and PBR optimization, respectively. The subscripts "A" and "B" in CNRs and PBRs below correspond to "a. CNR optimization" and "b. PBR optimization", respectively.

a. CNR optimization

Through optimization, we find that maximum CNR is achieved when the intensity threshold is set at double of the mean intensity, that is $I_{\mathrm{t}} = 2\mu$. In this case, the CNR given by Supplementary Equation (5) becomes

$$\mathrm{CNR_{P\_A}} = \frac{2}{e}\sqrt{N}, \tag{7}$$

and the PBR given by Supplementary Equation (6) becomes

$$\mathrm{PBR_{P\_A}} = 3. \tag{8}$$

In this case, PBR decouples from CNR and is a constant independent of the number of summed speckle patterns. In our experiment (Fig. 3c in the article, $\tau = 500$ ms), we achieved a PBR of ~2.5.

b. PBR optimization

To maximize PBR, one would set I_{t} as high as possible as indicated by Supplementary Equation (6). However, the maximum I_{t} is bounded by the requirement that on average one speckle pattern is selected during display. This requirement can be described as $\alpha N = 1$. Substituting α with Supplementary Equation (2), we find that the intensity threshold for PBR optimization is given by $I_{\mathrm{t}} = \mu \ln N$. Substituting this equation into Supplementary Equation (6), we have the maximum PBR:

$$\mathrm{PBR_{P\_B}} = 1 + \ln N. \tag{9}$$

Using this intensity threshold to calculate CNR based on Supplementary Equation (5), we have

$$\mathrm{CNR_{P\_B}} = \ln N. \tag{10}$$

Supplementary Equations (9) and (10) show that the PBR and CNR are coupled in this case. This relationship, PBR = CNR+1, is the same as that in wavefront shaping because in both cases the intensity

distribution of the background follows speckle intensity distribution where its mean intensity equals to its standard deviation.

Supplementary Fig. 5 plots CNR and PBR as a function of the number of measured optical modes. From this figure, we can find that in CNR optimization mode, CNR increases as a function of number of optical modes while the PBR remains the same. The enhancement in CNR means that the optical spot is more evident, which is the key metric to evaluate the ability of an imaging technique. In contrast, PBR fails to indicate this ability in this mode as it remains constant over the number of optical modes. In the PBR optimization mode, PBR and CNR are coupled and thus both of them can be used to evaluate the performance of an imaging technique.

Effect of shot noise

As the number of photons per speckle on each speckle pattern becomes lower, the photon shot noise becomes considerable. For simplicity, this section uses the same notation μ and other notations that are used to denote intensity in the previous section, but we use them here to indicate the numbers of photons within the time window of interest per speckle grain. The combination of the speckle noise and the shot noise can be calculated based on the total variance law:

$$\sigma_{\mathrm{t}}^2 = \alpha N \mu^2 + \alpha N \mu \, , \tag{11}$$

where the two terms on the right side are the variance of the speckle noise and the variance of the shot noise, respectively. Using the total noise to calculate the CNR, we have

$$\mathrm{CNR}_{\mathrm{P}}^{\mathrm{T}} = \frac{\overline{I_{\mathrm{sp}}} - \overline{I_{\mathrm{sb}}}}{\sigma_{\mathrm{t}}} = \frac{\alpha N I_{\mathrm{t}}}{\sqrt{\alpha N \mu^2 + \alpha N \mu}} = \sqrt{N} \exp\left(-\frac{I_{\mathrm{t}}}{2\mu}\right) \frac{I_{\mathrm{t}}}{\sqrt{\mu^2 + \mu}} \, . \tag{12}$$

Similar to the derivation for Supplementary Equation (7), the maximum CNR can be achieved by setting $I_{\mathrm{t}}=2\mu$, and Supplementary Equation (12) becomes

$$\mathrm{CNR}_{\mathrm{P\_A}}^{\mathrm{T}} = \frac{2}{e} \sqrt{N} \frac{1}{\sqrt{1 + \frac{1}{\mu}}} \, . \tag{13}$$

Compared to Supplementary Equation (7), we find that the shot noise becomes considerable versus the displayed pattern when the average number of photons per speckle within the time window of interest is on the order of one or less. Supplementary Fig. 6 shows the relation between CNR and N at different levels of

shot noise based on Supplementary Equation (13). In our experiment (Fig. 3c in the article) the light intensity on the background is about 3.2×10^5 photons per ms per speckle.

From Supplementary Equation (11), we note that the variance of the background consists of speckle noise and shot noise, and the noise terms determine the signal-to-noise ratio (SNR) of the optical spot. When the photon budget is high enough that the shot noise is negligible to the speckle noise, the SNR of the optical spot is more closely related to the CNR. As the photon budget becomes lower, the shot noise dominates the speckle noise, and the SNR of the optical spot becomes more closely related to the PBR since the shot noise directly results from the fluctuation of the background.

Null energy point

For an OCIS generated null energy point, the portion of speckle patterns being selected is given by

$$\alpha=\int_0^{I_\mathrm{t}}\frac{1}{\mu}\exp\left(-\frac{I}{\mu}\right)dI=1-\exp\left(-\frac{I_\mathrm{t}}{\mu}\right). \tag{14}$$

In this case, the expected intensity of the point of interest among the selected patterns is given by

$$\overline{I_\mathrm{n}}=\frac{1}{\alpha}\int_0^{I_\mathrm{t}}I\times P(I)dI\ . \tag{15}$$

By substituting the probability density function $P(I)$ with Supplementary Equation (1), we have

$$\overline{I_\mathrm{n}}=I_\mathrm{t}-\frac{I_\mathrm{t}}{1-\exp\left(-\frac{I_\mathrm{t}}{\mu}\right)}+\mu \tag{16}$$

If the system measures N speckle patterns in total, the number of selected speckle patterns is αN, and therefore the expected intensity of the sum of these patterns at the null energy point is given by $\overline{I_\mathrm{sn}}=\alpha N\overline{I_\mathrm{n}}$. Likewise, the expected intensity of the sum of these patterns at the background is given by $\overline{I_\mathrm{sb}}=\alpha N\mu$, and the standard deviation of the background is $\sigma_\mathrm{sb}=\sqrt{\alpha N}\sigma=\sqrt{\alpha N}\mu$. Therefore, the CNR, which is defined as the ratio between the background-subtracted null intensity and the standard deviation of the background, is given by

$$\mathrm{CNR_N} = \frac{\overline{I_{\mathrm{sn}}} - \overline{I_{\mathrm{sb}}}}{\sigma_{\mathrm{sb}}} = -\sqrt{N}\,\frac{I_{\mathrm{t}}}{\mu}\,\frac{\exp\left(-\frac{I_{\mathrm{t}}}{\mu}\right)}{\sqrt{1-\exp\left(-\frac{I_{\mathrm{t}}}{\mu}\right)}}\,. \tag{17}$$

The PBR, which is defined as the ratio between the negative peak or null point intensity and the mean of the background, is given by

$$\mathrm{PBR_N} = \frac{\overline{I_{\mathrm{sn}}}}{\overline{I_{\mathrm{sb}}}} = 1 - \frac{I_{\mathrm{t}}/\mu}{1-\exp\left(-\frac{I_{\mathrm{t}}}{\mu}\right)} + \frac{I_{\mathrm{t}}}{\mu}\,. \tag{18}$$

Supplementary Note 2 | Comparison of CNR between feedback based OCIS and feedback based wavefront shaping.

For optical spot pattern formed by OCIS, the optimal CNR is achieved when $I_{\mathrm{t}} = 2\mu$ based on Supplementary Equation (5). In this case, $\mathrm{CNR} = \frac{2}{e}\sqrt{N}$. In our experiment, the maximum rotating angle (θ) of the galvo mirror is 30°, and the diameter (d) of the laser beam is ~2 mm. The divergence angle ($\Delta\theta$) of the beam due to diffraction is approximately $\Delta\theta = \lambda / d = 2.6\times10^{-4}\,rad = 0.015^{\circ}$, where the optical wavelength λ is 532 nm. Then the number of independent optical modes (N) is $N = \theta / \Delta\theta = 2\times10^{3}$. Therefore, the number of controllable modes per unit time for intensity modulation (C_{OCIS}) is $C_{\mathrm{OCIS}} = N / T = 8\times10^{3}\,\mathrm{ms}^{-1}$, where T is the resonant period of the galvo mirror (250 μs). Therefore, for OCIS, the relationship between $\mathrm{CNR_{OSIS}}$ and operation time (t) is given by $\mathrm{CNR_{OCIS}} = \frac{2}{e}\sqrt{C_{\mathrm{OCIS}}t}$, which is plotted in the blue curve in Supplementary Fig. 7a.

As a reference, we analyze the performance of a typical feedback-based wavefront shaping method, which was demonstrated by Conkey, et al.[2] using a DMD-based setup. Their system was able to control 256 optical modes in 33.8 ms and provided a theoretical PBR ($\mathrm{PBR_{WS}}$) of 256 approximately. Therefore the number of controllable modes per unit time of this wavefront shaping method (C_{WS}) is equal to $C_{\mathrm{WS}} = 256/33.8\mathrm{ms} = 7.6\mathrm{ms}^{-1}$. Because PBR is linear as a function of the number of controllable modes and thus the required operation time, the relationship between PBR and operation time (t) can be approximated

to the equation $\mathrm{PBR_{WS}} = C_{\mathrm{WS}}t$, $\left(t \mid C_{\mathrm{WS}}t > 1\right)$. The theoretical CNR of wavefront shaping ($\mathrm{CNR_{WS}}$) is given by $\mathrm{CNR_{WS}} = \mathrm{PBR_{WS}} - 1$, provided that the standard deviation of the fully developed speckle pattern is equal to its mean. Therefore, the relationship between CNR of wavefront shaping and operation time is $\mathrm{CNR_{WS}} = C_{\mathrm{WS}}t - 1$, $\left(t \mid C_{\mathrm{WS}}t > 1\right)$, which is plotted in red in Supplementary Fig. 7a.

The comparison shown in Supplementary Fig. 7a implies that the feedback-based OCIS outperforms the feedback-based wavefront shaping using DMD in the regime where the operation time is shorter than ~80 ms. This advantage is critical because the decorrelation time of biological tissue *in vivo* can be less than one millisecond[3]. To simulate the CNR given a sample with a decorrelation time of 1 ms, we multiplied the CNR functions in Supplementary Fig. 7a with a negative exponential correlation function with a decay constant of 1 ms. Supplementary Fig. 7b plots the CNR as a function of time considering the effect of sample decorrelation. The advantage of high CNR enhancement at short time scale becomes prominent.

Supplementary Note 3 | Discussions on the security of OCIS-based communications

There are two typical scenarios where light can be received by a third party (Supplementary Fig. 9). In free space, scatterers, such as dust, fog, turbid water, or opaque walls, scatter light outside the line-of-sight of the communication parties. In waveguide geometry, leaky modes allow the light to be coupled out of the waveguide. There is also an extreme case where an optical fiber waveguide is cut and a beam splitter is inserted in between. Although this type of interception can be easily detected, we also include it in our security analysis framework.

Without OCIS, light scattering and waveguide leakage will allow a third party to receive the same copy of the information as the primary communication parties. In this case, the transmitted information can only be secured by the use of a digital key to encrypt the information. If the third party hacks the digital key, the information would be compromised.

OCIS provides a physical layer of encryption, which can be used on top of digital encryption. Here we analyze the probability of decoding the OCIS encrypted information by coupling and detecting the light during propagation in the aforementioned scenarios. In principle, if the third party (Chuck) can measure the full optical field from the primary communication parties (Alice and Bob), he can decode the information by correlating the two optical fields based on the time-reversal symmetry of light propagation. In practice, measuring the optical field in the middle of the scattering media is extremely challenging in OCIS for several reasons. First, measuring the full field requires a full coverage in free space or cutting the optical fibers, which can be easily monitored as discussed above. Second, OCIS can use multiple spatially incoherent light sources, between which there is no static phase difference, to prevent phase measurement.

Therefore, we would like to focus on a more practical case where only the intensity patterns are partially measured by the third party during the transmission.

The process by which Chuck can make such an intensity pattern measurement is as follows. First, Alice sends a single-mode laser pulse through the scattering media to establish a channel map with Bob. Chuck measures a speckle pattern in the middle of the scattering medium, and Bob measures a speckle pattern on the other end of the scattering medium. For simplicity, here we analyze the case where Bob only sends light through one channel for one bit of information transmission. This channel is randomly selected from the channels that meet the intensity requirement and the scattering medium is refreshed when all the channels have been used. Chuck measures the second speckle pattern in the middle when Bob sends one bit back to Alice. In this case, Chuck can try to decode the information by calculating the sign of the correlation coefficient between the speckle patterns.

Mathematically, we can explicitly calculate the correlation coefficient C of the intensity patterns measured by Chuck and analyze its expected value and the standard deviation. The correlation coefficient C has the form of

$$C = \frac{\frac{1}{M_0}\sum_{r=1}^{M_0}\left(I_{\mathrm{C,A}}(r) - \bar{I}_{\mathrm{C,A}}\right)\left(I_{\mathrm{C,B}}(r) - \bar{I}_{\mathrm{C,B}}\right)}{\bar{I}_{\mathrm{C,A}}\bar{I}_{\mathrm{C,B}}}, \tag{19}$$

where $I_{\mathrm{C,A}}(r)$ and $I_{\mathrm{C,B}}(r)$ are the intensity patterns measured by Chuck when Alice and Bob send the light pulses, respectively; $\bar{I}_{\mathrm{C,B}}$ and $\bar{I}_{\mathrm{C,A}}$ are the mean intensities of these two patterns, respectively; M_0 is the total number of spatial modes generated by the scattering medium and is much larger than one; r is the index of the speckle grains. After mathematical derivation based on the complex field relationship ensured by reciprocity, the expected value of correlation coefficient C has the following expression:

$$\mathrm{E}(C) \approx \frac{I_{\mathrm{t}} - \bar{I}}{M_0 \bar{I}}, \tag{20}$$

where I_{t} is the intensity of the speckle grain that Bob selects as the channel to send one bit back to Alice; $\bar{I}$ is the mean intensity of the speckle grains at Bob's side. For simplicity, here we assume that Alice and Bob use the same amount of power for the laser pulses they send to each other. In this case, the speckle power that Alice observes is also I_{t}, the same as that of the speckle that Bob selects based on the intensity transmission matrix theory.

Supplementary Equation (20) has an intuitive interpretation. The numerator $I_{\mathrm{t}}-\bar{I}$ indicates the power deviated from the mean power at the mode of interest that Alice observes or Bob selects. If Bob randomly picks a channel to send light back to Alice, the expected value of this deviation should be zero, and the expected correlation between Chuck's patterns would also be zero. Therefore, the expected value of the correlation coefficient describes the energy ratio between the part that is deviated from mean at the mode of interest and the total energy.

For each bit that is transmitted, Chuck can calculate the correlation between the two speckle patterns and obtain one correlation coefficient C. Therefore, it is also important to know the deviation of the one-time calculation from the expected value of the correlation coefficient C. The error or the standard deviation of the correlation coefficient is given by[4]

$$\mathrm{std}(C)\approx\sqrt{\frac{1}{M}}, \tag{21}$$

where M is the number of modes that Chuck measures out of the M_0 modes carried by the scattering medium. Here we assume that the measurement is well above shot noise limit. Therefore, the SNR of the information that Chuck obtained is given by

$$\mathrm{SNR}_{\mathrm{C}}=\left(\frac{\mathrm{E}(C)}{\mathrm{std}(C)}\right)^2=\left(\frac{I_{\mathrm{t}}-\bar{I}}{\bar{I}}\right)^2\frac{M}{M_0^2}. \tag{22}$$

Here we provide an example calculation of the SNR that Chuck may receive. Let us assume that a scattering medium carries 10^6 modes (M_0) and Chuck measures all the modes in an extreme case (M=M_0); the mean of the threshold that Bob chooses is $2\bar{I}$. In this case, the SNR of the correlation coefficient C is ~10^{-6}, which implies that it is very difficult for Chuck to obtain meaningful information. In practice, Chuck can only measure a small portion of the modes, which results in an even lower SNR. The leakage of information can be further mitigated by the combination of digital encryption, such as leakage-resilient cryptography[5].

By providing a physical layer of encryption, OCIS based secure communication can potentially be applied to several scenarios including free-space and fiber-based communication. Importantly, this physical encryption is complementary to and able to work with key based encryption, which includes keys that are generated with optical approaches such as quantum key distribution[6]. Compared to quantum key distribution, OCIS does not have a strict requirement on the number of photons used in communications as long as Alice and Bob can measure sufficient photons. It should be noted that OCIS requires multimode fibers to provide the physical encryption, which is likely to be a limiting factor for immediate use in some

existing networks that are based on single mode fibers. In our experiment, the data transfer rate is limited by the refreshing rate of the DMD. The data transfer rate can possibly be improved by using an acousto-optic deflector (AOD) to select the intensity channels in the future.

Supplementary Methods

Image transmission through scattering media with OCIS

With the knowledge of intensity mapping between the input plane and target plane, OCIS is able to correct for disordered scattering and allow for direct transmission of intensity information through scattering media. Here, we demonstrate this ability by directly imaging an object through a scattering medium. From the recording process of optical intensity transpose (Fig. 4c or Supplementary Fig. 8a1), we can obtain a map of optical channels between the input plane and target plane during a galvo mirror scan. We can then direct the light from the high-throughput channels to a point on the detector plane during the second galvo scan. In this case, we modulate and send light to the high throughput channels sequentially when the galvo mirror rotates to positions where the channels are connected to the point (Supplementary Fig. 8a1). As such, we obtain a time-averaged optical spot on the detector plane as a PSF of the imaging system.

To form a wide field image through the scattering medium, here we utilize the tilt-tilt correlation or angular memory effect of a thin scattering medium[7,8]. Within an angular memory effect range, tilting of an input wavefront to a scattering medium causes tilting of the scattered output wavefront, and these two optical wavefronts remain highly correlated. For a thin scattering medium, the correlation is maintained within a reasonable tilting angle for wide field imaging. Therefore, the modulation signal that generates the PSF is also a valid solution to cast a neighboring spot on the target plane to a shifted optical spot on the detector plane through the scattering medium (Supplementary Fig. 8a2 and 8b2). The method maps to the phase compensation approach that enables wide field imaging through thin scattering medium in optical wavefront shaping[8,9]. In both cases, we can interpret the system as a piece of compensation optics that corrects for the scattering of the sample and that allows us to see through the scattering medium directly (Supplementary Fig. 8c). Intriguingly, no phase information or manipulation is required for OCIS to compensate for the optical scattering here.

To directly correct optical scattering and form an image in free space through a thin scattering medium experimentally, we need to first calibrate the scattering medium by measuring the response of a point source on the target plane through the scattering medium (Supplementary Fig. 8a1). See Supplementary Fig. 2d for more details on the setup. This step is the same as the recording process in optical intensity transpose. We then used a target consisting of two points near the calibration point with a separation of 20 μm (Supplementary Fig. 8a2). To image the object, the laser source was modulated with the signal measured

from the calibration step as the galvo mirror scans. We placed a camera with an exposure time covering the galvo scan duration to directly observe the image of the two spots on the detector plane. As shown in Supplementary Fig. 8d, the image information directly was transmitted through the scattering medium with OCIS.

The optical setup of realizing imaging through scattering media is shown in Supplementary Fig. 2d. The initial focus for the intensity response measurement as well as the two-point object pattern for imaging was created by using an SLM. During measurement, the SLM displayed a focus on the target plane and the PMT measured the speckle intensity signal as the galvo mirror was scanning. During wide-field imaging, the SLM displayed two foci around the original focus as a target. To directly image this target, we repeated the galvo mirror scanning and modulated the intensity of the laser illumination using the AOM based on the recorded intensity response. The camera was used to directly observe the image of the two foci through the scattering medium.

It should be noted that the two spots here are coherently illuminated and interfere with each other during each optical pulse. Although the light field of a focus on the image plane interferes with the background light field from the other spot, their phase relationship is random between pulses. Therefore, the expected intensity of the focus after integrating many pulses remains the same as the case of one spot. However, both the mean intensity and standard deviation of the background are doubled due to the interference between the two spots. As the number of spots increases, the CNR of the image decreases linearly. To obtain a higher CNR for a complex object, we can consider using a temporally coherent (or narrow band) and spatially incoherent light source, e.g. using a rotating diffuser to scramble the phase of a coherent source. In this case, the light is coherent within one spot but not between two spots. As a result, the standard deviation of the background would be proportional to the square root of the number of spots.

References

1. Goodman, J. W. *Speckle phenomena in optics: theory and applications*. *Book* (Roberts and Company Publishers, 2007).
2. Conkey, D. B., Caravaca-Aguirre, A. M. & Piestun, R. High-speed scattering medium characterization with application to focusing light through turbid media. *Opt Express* **20**, 1733–1740 (2012).
3. Durduran, T., Choe, R., Baker, W. B. & Yodh, A. G. Diffuse optics for tissue monitoring and tomography. *Reports on Progress in Physics* vol. 73 076701 (2010).
4. Student. Probable error of a correlation coefficient. *Biometrika* **6**, 302–310 (1908).
5. Kalai, Y. T. & Reyzin, L. *A Survey of Leakage-Resilient Cryptography*. https://eprint.iacr.org. (2019).
6. Bennett, C. H., Bessette, F., Brassard, G., Salvail, L. & Smolin, J. Experimental quantum

cryptography. *J. Cryptol.* **5**, 3–28 (1992).

7. Freund, I., Rosenbluh, M. & Feng, S. Memory Effects in Propagation of Optical Waves through Disordered Media. *Phys. Rev. Lett.* **61**, 2328–2331 (1988).

8. Katz, O., Small, E. & Silberberg, Y. Looking around corners and through thin turbid layers in real time with scattered incoherent light. *Nat. Photonics* **6**, 549–553 (2012).

9. Katz, O., Heidmann, P., Fink, M. & Gigan, S. Non-invasive single-shot imaging through scattering layers and around corners via speckle correlations. *Nat. Photonics* **8**, 784–790 (2014).
